# Endoscopic treatment of colorectal anastomotic leakage with an innovative hybrid vacuum stent

**DOI:** 10.1055/a-2974-6039

**Published:** 2026-10-22

**Authors:** Ivo Mendes, Carolina Palma, Carolina Renda Rodrigues, Susana Alexandra Henriques, Ana Maria Monteiro, Gonçalo Nunes

**Affiliations:** 1 Gastroenterology Department 70816 Hospital Garcia de Orta EPEAlmadaSetubalPortugal; 2 Egas Moniz Center for Interdisciplinary Research (CiiEM) 112055 Egas Moniz School of Health and ScienceCaparicaSetubalPortugal; 3 Surgery Department 70816 Hospital Garcia de Orta EPEAlmadaSetubalPortugal

A 73-year-old man underwent laparoscopic rectal anterior resection due to an adenocarcinoma (pT2N0M0). In the early postoperative period, he developed fever and abdominal pain. Computed tomography (CT) with rectal contrast revealed extravasation from the colorectal anastomosis into the retroperitoneum. Endoscopic evaluation confirmed a 10-mm defect located 16 cm from the anal verge, with congested and friable margins adjacent to surgical clips ( Fig. 1 ). Endoscopic treatment with a 80×24 mm hybrid vacuum stent (VACStent GI Colon) was proposed ( Video 1 ). A marking clip was placed at the level of the anastomosis, and a 0.035-inch guidewire was passed into the descending colon. After saline irrigation, the delivery system was advanced over-the-wire, and the stent was successfully deployed under fluoroscopic control, centered on the clip ( Figs. 2 3 ). The suction catheter was connected to a vacuum pump at − 120 mm Hg. Saline irrigation (40 mL) through the drainage tube was performed three times/d. The patient also underwent laparoscopic peritoneal lavage and creation of a protective ileostomy to improve sepsis control.

**Fig. 1:**
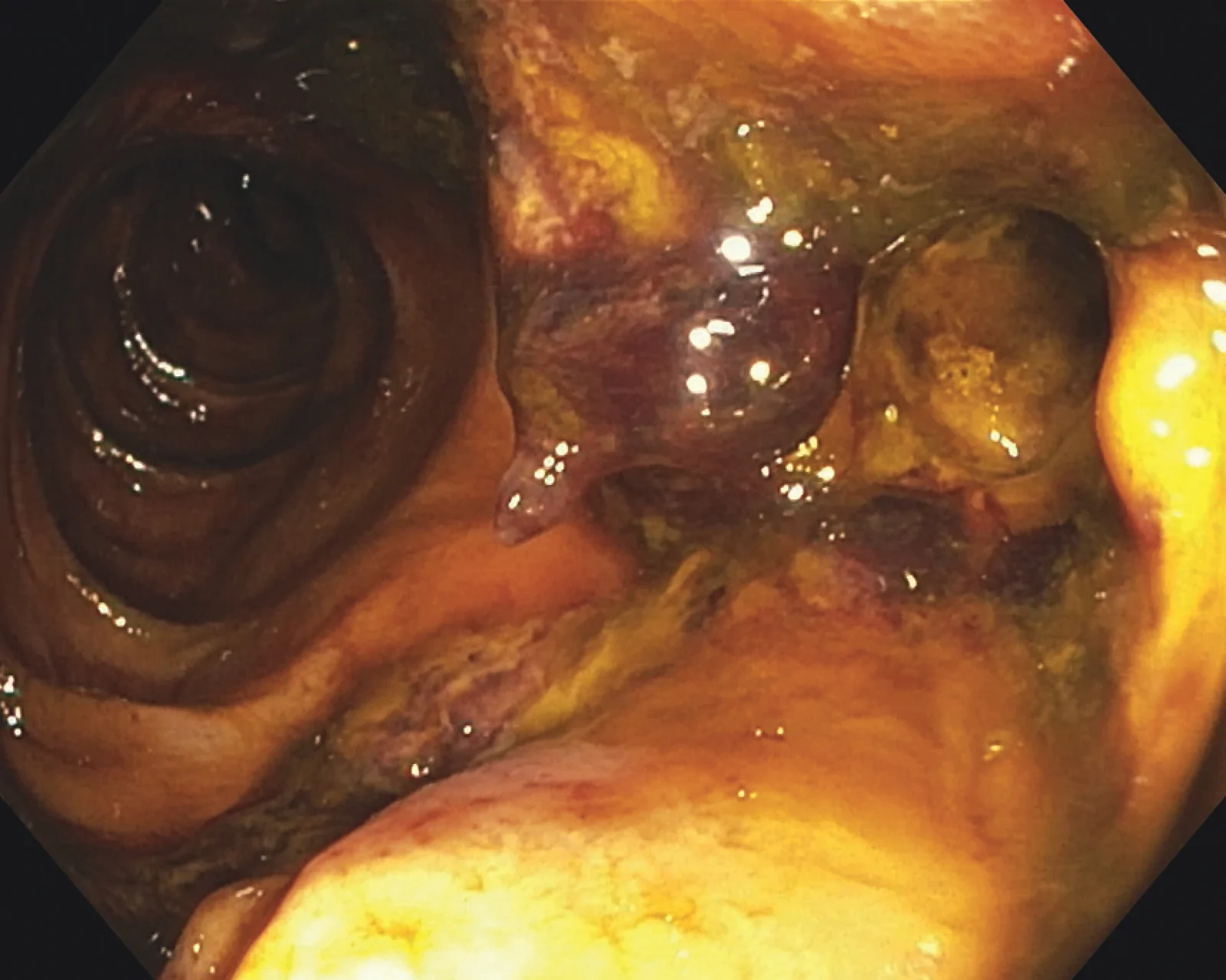
Endoscopic evaluation confirmed a 10-mm defect located 16 cm from the anal verge, with congested and friable margins adjacent to surgical clips.

**Video 1** Endoscopic treatment of colorectal anastomotic leakage with the VACStent. The video link below: URI: .


**Fig. 2:**
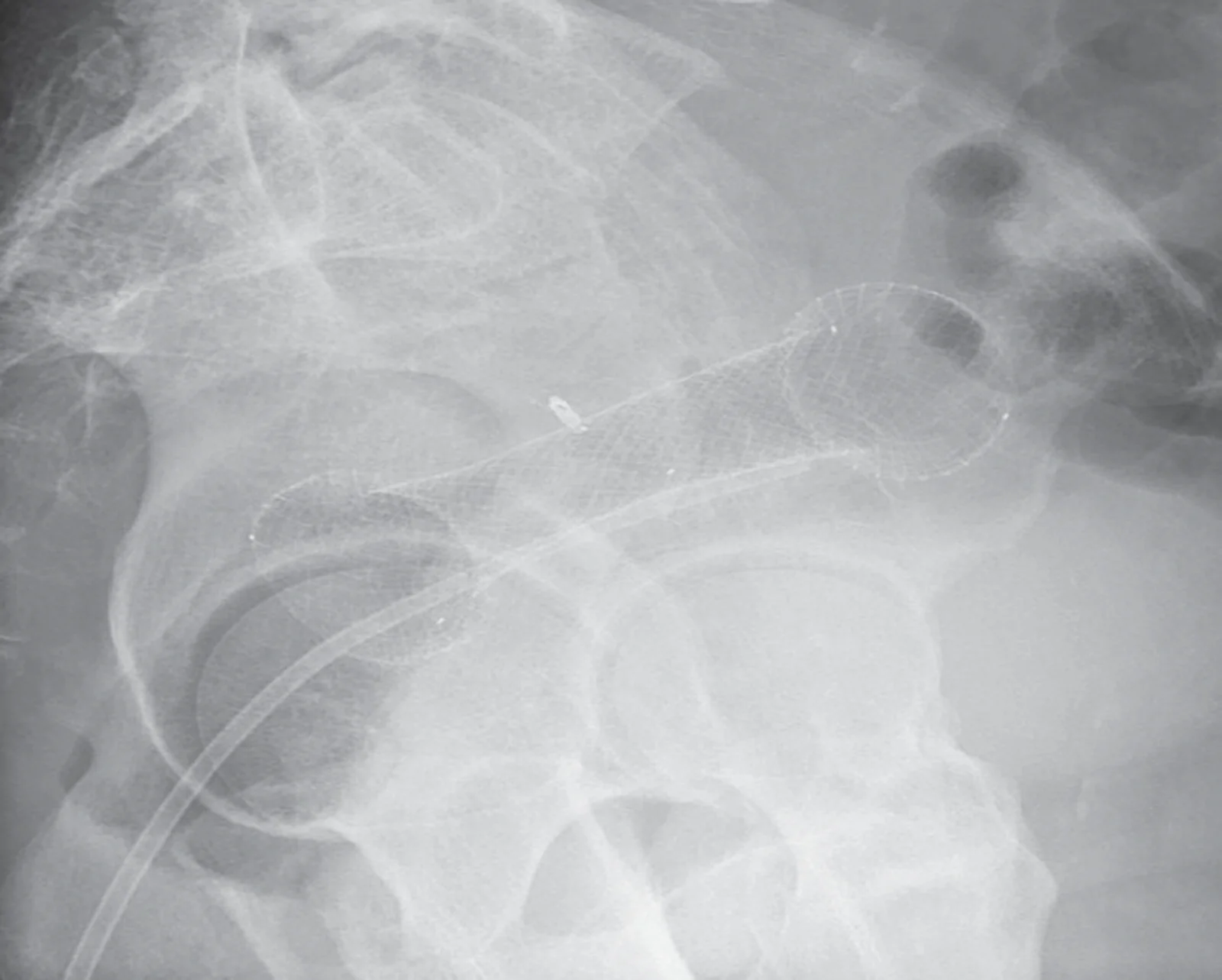
The stent was successfully deployed, centered on the clip (a fluoroscopic image).

**Fig. 3:**
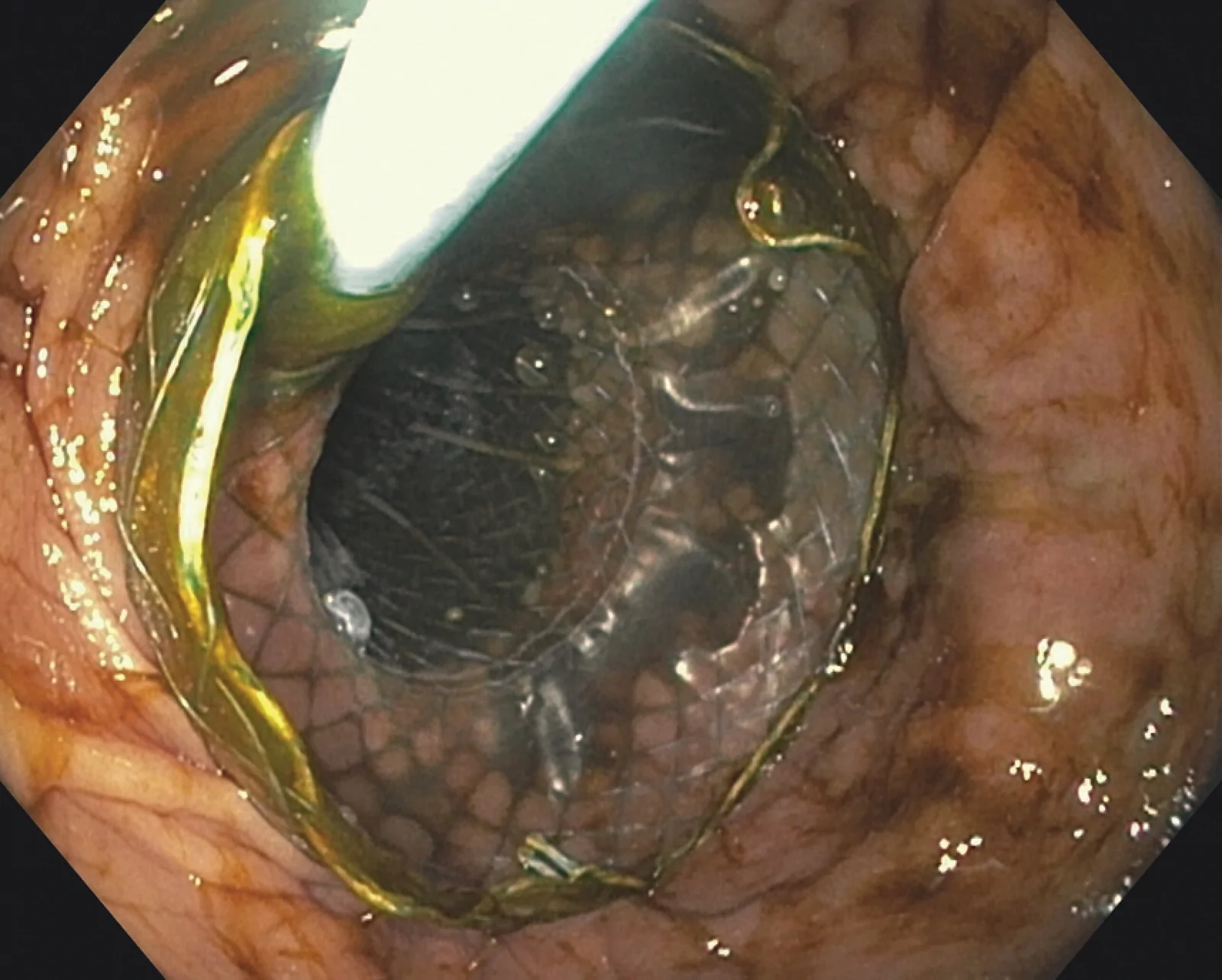
The stent was successfully deployed, centered on the clip (an endoscopic image).

Stent removal was scheduled after 7 days with the vacuum discontinued the day before while maintaining saline irrigation. The stent was easily retrieved by grasping its distal end with biopsy forceps, revealing extensive granulation tissue ( Fig. 4 ). Although no contrast extravasation was observed, complete closure of the defect could not be confidently ensured, and a second vacuum stent was placed for more 7 days. In addition, percutaneous drainage of a retroperitoneal collection was performed. After removal of the second stent, complete closure of the defect was confirmed endoscopically ( Fig. 5 ) and on follow-up CT. Bowel continuity was restored after 10 days and the patient was discharged without symptoms.

**Fig. 4:**
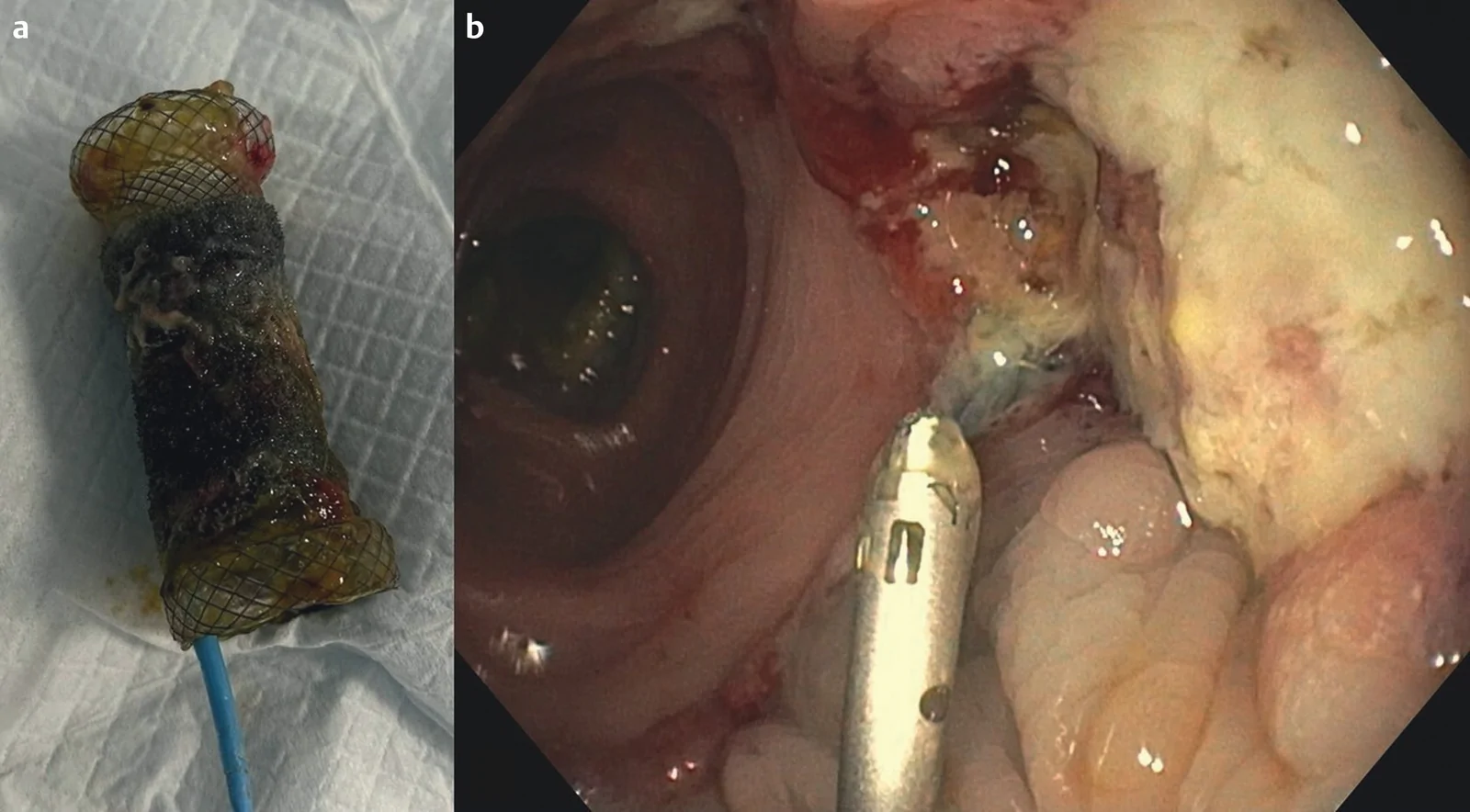
( **a** ) The vacuum stent after removal. ( **b** ) Extensive granulation tissue after removal of the first stent.

**Fig. 5:**
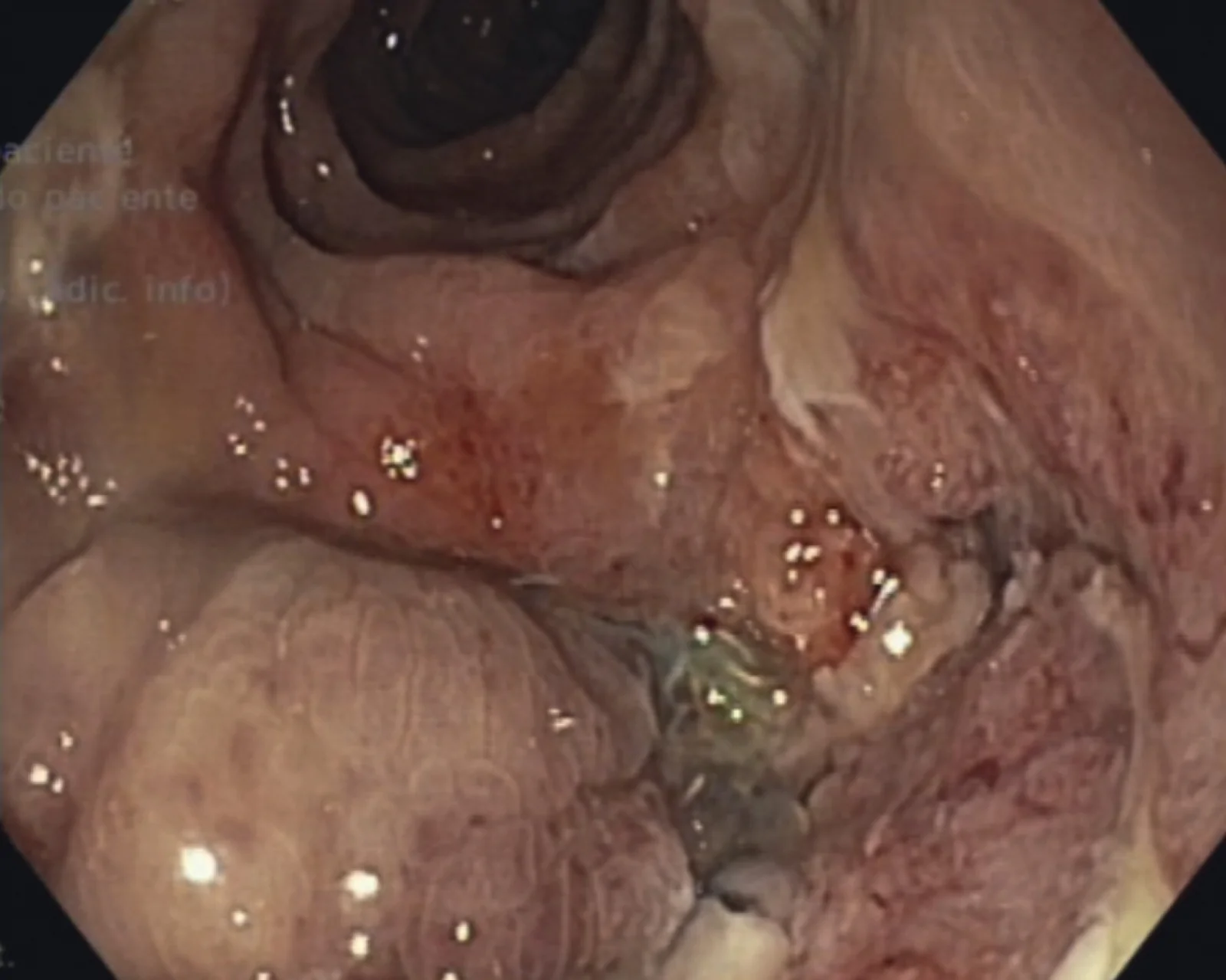
Complete closure of the defect after removal of the second stent.

The VACStent combines the advantages of covered metal stents and endoscopic vacuum therapy allowing direct defect closure, drainage of secretions and healing promotion, while maintaining luminal patency and avoiding migration. This case demonstrates its effectiveness for the treatment of colorectal leaks.

Endoscopy_UCTN_Code_CPL_1AJ_2AH

## Declaration of genAI Use

The authors confirm that no generative artificial intelligence was used in the preparation of this manuscript.

